# Osthole Activates FGF21 Expression by Mediating Activation of ATF4 in Human Hepatocyte HepG2 Cells

**DOI:** 10.3390/ijms27021003

**Published:** 2026-01-19

**Authors:** Akishi Taguchi, Masaya Araki, Tomoya Yamashita, Ryo Kanazawa, Itsuki Terao, Kyohei Suzuki, Yuhei Tsuchimoto, Takashi Matsuzaka, Hirohito Sone, Hitoshi Shimano, Yoshimi Nakagawa

**Affiliations:** 1 Division of Complex Biosystem Research, Department of Research and Development, Institute of Natural Medicine, University of Toyama, Toyama 930-0194, Japan; s2160232@ems.u-toyama.ac.jp (A.T.); maraki@inm.u-toyama.ac.jp (M.A.); s2160255@ems.u-toyama.ac.jp (T.Y.); s2260307@ems.u-toyama.ac.jp (R.K.); m25b1313@ems.u-toyama.ac.jp (I.T.); kyo2000he48i@gmail.com (K.S.); udonbad@icloud.com (Y.T.); 2 Department of Endocrinology and Metabolism, Faculty of Medicine, University of Tsukuba, Ibaraki 305-8575, Japan; t-matsuz@md.tsukuba.ac.jp (T.M.); hshimano@md.tsukuba.ac.jp (H.S.); 3 Transborder Medical Research Center (TMRC), University of Tsukuba, Ibaraki 305-8575, Japan; 4 Department of Hematology, Endocrinology and Metabolism, Faculty of Medicine, Niigata University, Niigata 951-8510, Japan; sone@med.niigata-u.ac.jp

**Keywords:** osthole, FGF21, ATF4, coumarin derivative, gene expression, signal transduction

## Abstract

Osthole is a natural coumarin derivative found in several medicinal plants, including *Cnidium monnieri* and *Angelica pubescens*. It has been studied for its various biological properties, such as anti-inflammatory, neuroprotective, osteogenic, cardioprotective, antimicrobial, and antiparasitic effects. Osthole was found to induce Fibroblast growth factor 21 (*FGF21*) expression. Among the known transcription factors that regulate FGF21 induction, activating transcription factor 4 (*ATF4*) expression was found to be upregulated by osthole. Additionally, as osthole induced ATF4 downstream gene expression, it was concluded that it activates ATF4 signaling. ATF4 knockdown significantly suppressed osthole-mediated induction of *FGF21* expression. These findings suggest that osthole activates *FGF21* expression via ATF4 activation.

## 1. Introduction

Metabolically-dysfunction-associated fatty liver disease (MAFLD) affects approximately 20–30% of the global population, making it one of the most prevalent causes of chronic liver disease [1]. Among patients with metabolic dysfunction-associated steatohepatitis (MASH), nearly 50–60% experience comorbidities such as diabetes mellitus, cardiovascular disease, and hyperlipidemia [1]. MAFLD and MASH both show strong correlations with conditions like insulin resistance and obesity [2]. MAFLD is recognized as a “multi-hit” condition [3], resulting from a complex interplay of mechanisms including insulin resistance, oxidative stress, mitochondrial dysfunction, endoplasmic reticulum stress, and apoptosis. Gaining a deeper understanding of these interconnected processes may offer valuable insights into MAFLD progression.

The fruits of *Cnidium monnieri* and *Angelica pubescens* have long been utilized in traditional Chinese medicine. Osthol, a key bioactive compound extracted from *Cnidium monnieri* and *Angelica pubescens*, exhibits both anti-inflammatory and hepatic fat-oxidizing properties. Osthole not only reduced fasting blood glucose levels and hepatic fat accumulation but also improved insulin resistance in rats with diet-induced obesity [4]. Osthole reduced the hepatic fat level by activating the hepatic gene expression of peroxisome proliferator-activated receptor α (PPARα) and PPARγ in rats [5]. In an alcoholic fatty liver disease mouse model, osthole activated superoxide dismutase, resulting in reduced oxidative stress [6].

FGF21 is primarily produced in the liver and released into the bloodstream, where it influences various peripheral tissues to regulate systemic nutrient metabolism. It reduces plasma glucose levels by increasing glucose uptake in adipose tissue [7]. It also plays a crucial role in regulating lipid metabolism and reducing lipid accumulation in adipose tissue through several mechanisms. It promotes fatty acid oxidation in adipose tissue, shifting metabolism toward energy expenditure rather than lipid storage [8]. It activates thermogenesis by inducing thermogenic gene expression and white adipose tissue browning, thereby increasing the levels of peroxisome proliferator-activated receptor gamma coactivator 1α (PGC-1α) [8]. FGF21 also plays a significant role in regulating food intake and macronutrient preference. FGF21 reduces the consumption of sweet foods and alcohol while boosting protein intake [9]. FGF21 is a key metabolic hormone that has been shown to ameliorate various lifestyle-related diseases, including obesity, type 2 diabetes, dyslipidemia, and fatty liver disease [10].

FGF21 improves the physiological characteristics of some mouse models of type 2 diabetes [7,11]; therefore, it could be a potential therapeutic target for metabolic diseases. *FGF21* gene expression is regulated by certain transcription factors. It is upregulated by PPARα [12] and cyclic adenosine monophosphate-responsive element-binding protein H (CREBH) [13] in the fasted state and by carbohydrate-responsive element-binding protein (ChREBP) [14] in the fed state. In addition, nuclear factor erythroid 2-related factor 2 (NRF2) [15] and endoplasmic reticulum (ER) stress transcription factors, including activating transcription factor 4 (ATF4) [16], ATF6 [17], and X-box binding protein 1s (XBP1s) [18], increase *Fgf21* expression.

ATF4, a key regulator of the Integrated Stress Response (ISR), plays a crucial role in ER stress adaptation [19]. ISR is triggered by various stressors, including amino acid deprivation, oxidative stress, and ER stress [19]. ATF4 influences lipid metabolism, amino acid transport, and mitochondrial function [19]. phosphorylated protein kinase RNA-like ER kinase (PERK) phosphorylates eukaryotic initiation factor 2α (eIF2α), leading to ATF4 induction [20]. ATF4 expression is upregulated by NRF2 [21], transcription factor E3 (TFE3), and transcription factor EB (TFEB) [22], *ATF4* expression is downregulated by CCAAT/enhancer-binding protein β (C/EBPβ) [23,24].

This study aimed to identify an activator of FGF21 in herbal medicines and their constituent components. We have identified osthole as a potential activator of *FGF21* expression in HepG2 cells.

## 2. Results

### 2.1. Osthole Induces FGF21 Expression in HepG2 Cells

**Figure 1:**
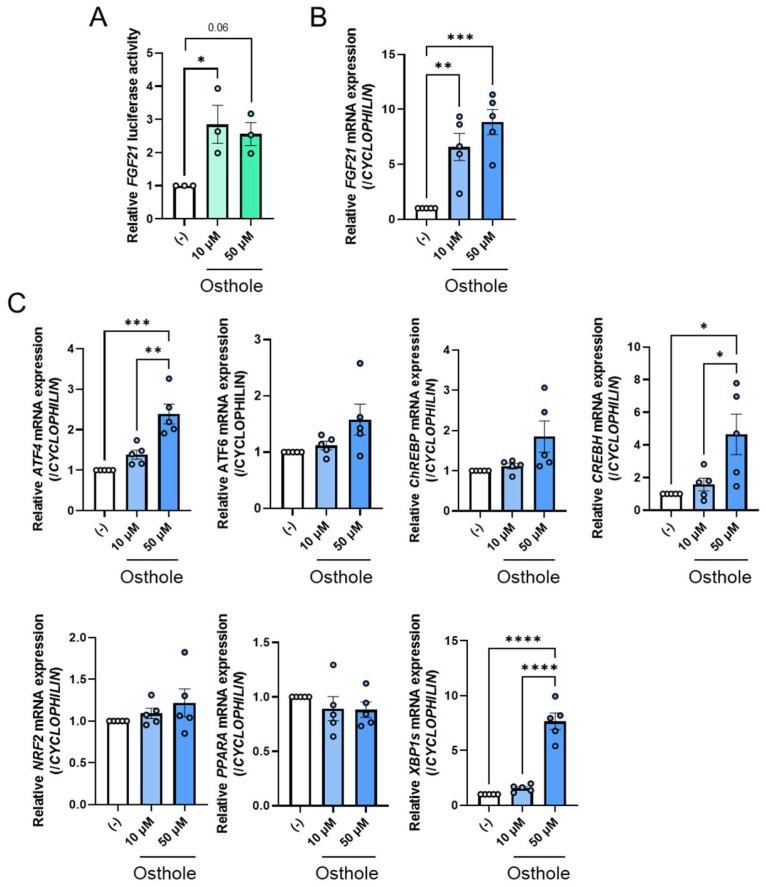
Osthole is identified as an activator of FGF21 expression in HepG2 cells. (**A**) The luciferase reporter assay for FGF21 promoter activity. HepG2 cells were co-transfected with pGL3-FGF21 and pRL-SV40 vectors. Twenty-four hours after transfection, cells were treated with 10 µM or 50 µM osthole or dimethyl sulfoxide (DMSO), which is indicated as (−), for an additional 24 h. *n* = 3 per group. (**B**) FGF21 gene expression levels in HepG2 cells treated with 10 µM or 50 µM osthole or DMSO, which is indicated as (−), for 24 h. *n* = 5 per group. (**C**) Expression of transcription factors involved in the regulation of FGF21 expression in HepG2 cells treated with 10 µM or 50 µM osthole or DMSO, which is indicated as (−), for 24 h. *n* = 5 per group. Data are presented as the mean ± SE. * *p* < 0.05; ** *p* < 0.01; *** *p* < 0.001; **** *p* < 0.0001. Statistical comparisons were performed using the one-way ANOVA, followed by Tukey’s post hoc test.

Since osthole has been reported to activate PPARα, a potential activator of FGF21, we hypothesized that it enhances FGF21 expression by activating PPARα. To investigate the effects of osthole on *FGF21* expression, we conducted a luciferase assay using pGL3-FGF21-transfected HepG2 cells, which belong to a human hepatoma cell line. As expected, osthole activated FGF21 luciferase activity at concentrations of 10 µM and 50 µM after 24 h compared to the control group (Figure 1A). Additionally, qPCR analyses confirmed that osthole significantly increased *FGF21* mRNA levels in HepG2 cells at concentrations of 10 µM and 50 µM after 24 h compared to the control group (Figure 1B). Osthole increased *FGF21* expression more efficiently than fenofibrate, a positive control for FGF21 activation (Figure S1). These findings suggest that osthole acts as a potential activator of FGF21 expression.

### 2.2. ATF4 Increased FGF21 Expression in Response to Osthole

**Figure 2:**
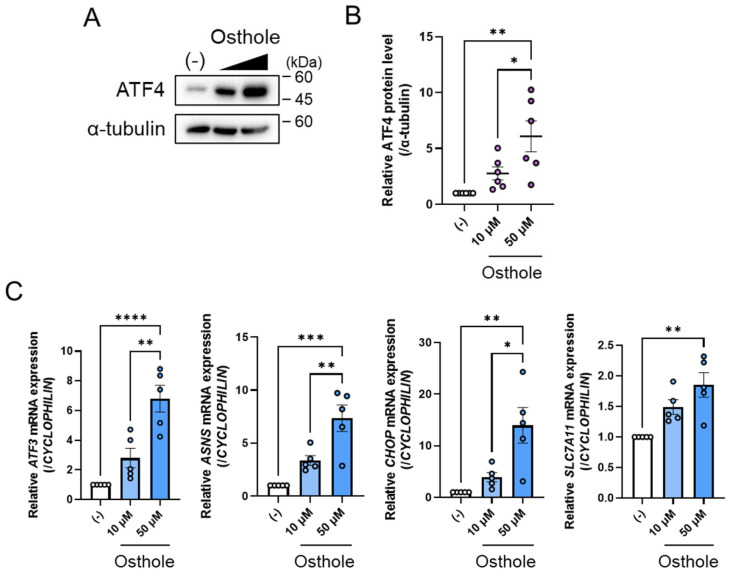
Osthole effects on ATF4 signaling in HepG2 cells (**A**,**B**) ATF4 protein levels in HepG2 cells treated with 10 µM or 50 µM osthole or DMSO, which is indicated as (−), for 24 h (**A**). Band intensities were quantified. *n* = 6 per group (**B**). (**C**) Expression levels of ATF4-regulated genes in HepG2 cells treated with 10 µM or 50 µM osthole or DMSO, which is indicated as (−), for 24 h. *n* = 5 per group. Data are presented as the mean ± SE. * *p* < 0.05; ** *p* < 0.01; *** *p* < 0.001; **** *p* < 0.0001. Statistical comparisons were performed using the one-way ANOVA, followed by Tukey’s post hoc test.

The expression of *Fgf21* is upregulated by certain transcription factors, including ATF4, ATF6, ChREBP, CREBH, NRF2, PPARα, and XBP1s. To identify a transcription factor that controls osthole-mediated *FGF21* expression, we examined the gene expression of these factors. Fifty micromolar osthole significantly increased ATF4 expression compared to the control, and this increase tended to be dose-dependent (Figure 1C). Fifty micromolar osthole significantly increased the expression of *ATF4*, *CREBH*, and *XBP1s* compared to the control group (Figure 1C). However, the expression of *ATF6* and *ChREBP* tended to increase with osthole administration, but this increase was not statistically significant (Figure 1C). *NRF2* and *PPARA* were unchanged (Figure 1C). In this study, we focused on ATF4. We also confirmed that osthole increased ATF4 protein levels (Figure 2A). Consistent with this, 50 μM osthole increased the expression of some of ATF4’s typical target genes, such as *ATF3*, asparagine synthetase (*ASNS*), CCAAT/enhancer-binding protein homologous protein (*CHOP*), and solute carrier family 7 member 11 (*SLC7A11*) [25], and these increments tended to be dose-dependent (Figure 2B). Taken together, these results suggest that ATF4 is an effective activator of osthole-mediated *FGF21* expression in HepG2 cells.

### 2.3. Osthole Does Not Change the Molecules to Activate ATF4 Expression

**Figure 3:**
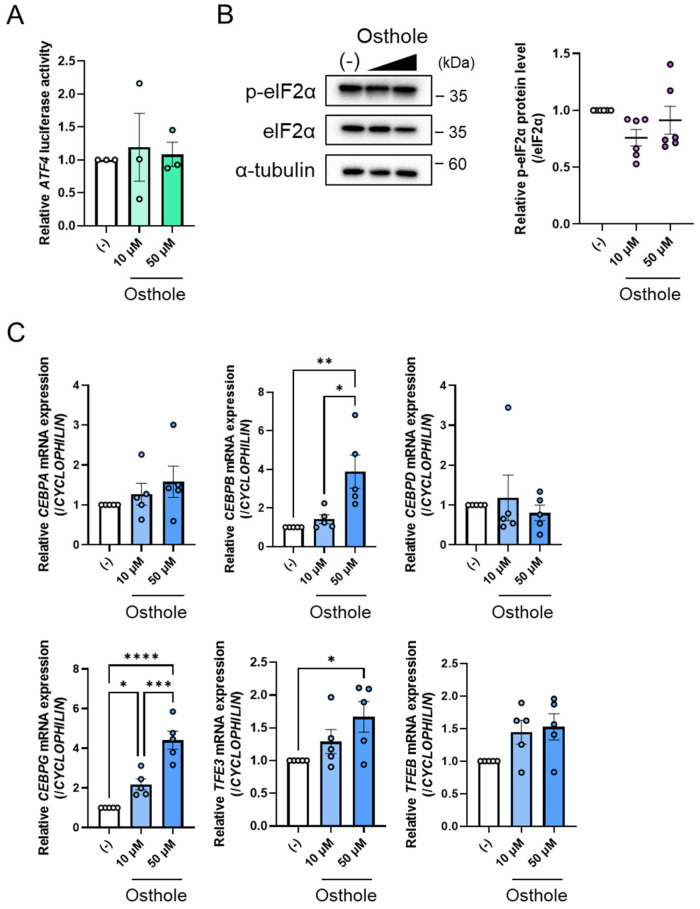
Osthole does not directly affect the promoter activity of ATF4 in HepG2 cells. (**A**) The luciferase reporter assay for ATF4 promoter activity. HepG2 cells were co-transfected with pGL3-ATF4 and pRL-SV40 vectors. Twenty-four hours after transfection, cells were treated with 10 µM or 50 µM osthole or DMSO, which is indicated as (−), for an additional 24 h. *n* = 3 per group. (**B**) Phosphorylated and total eIF2α protein levels in HepG2 cells treated with 10 µM or 50 µM osthole or DMSO, which is indicated as (−), for 24 h. Band intensities were quantified. *n* = 6 per group. (**C**) Expression levels of ATF4-regulated genes in HepG2 cells treated with 10 µM or 50 µM osthole or DMSO, which is indicated as (−), for 24 h. *n* = 5 per group. Data are presented as the mean ± SE. * *p* < 0.05; ** *p* < 0.01; *** *p* < 0.001; **** *p* < 0.0001. Statistical comparisons were performed using the one-way ANOVA, followed by Tukey’s post hoc test.

As osthole was found to increase the gene expression and protein levels of ATF4 (Figure 1C and Figure 2A), it was hypothesized that it might enhance ATF4 promoter activity. To test this theory, we performed a luciferase assay using a pGL3-ATF4 vector containing the *ATF4* promoter region from −0.5 kbp to −100 bp. However, osthole was found not to affect *ATF4* promoter activity (Figure 3A). Consequently, we investigated the factors that might regulate *ATF4* expression in response to osthole. The PERK-eIF2α signaling pathway increases ATF4 protein levels [26]. ER stress induces PERK, which activates eIF2α by phosphorylating it and eventually inducing ATF4 translation. Therefore, we determined the levels of phospho-eIF2α (p-eIF2α) and total eIF2α by Western blotting. We found that osthole did not alter the levels of p-eIF2α (Figure 3B), indicating that osthole does not affect PERK-eIF2α signaling pathway. Next, we examined transcription factors controlling *ATF4* expression, such as the C/EBP family, NRF2, TFE3, and TFEB. The expression of *CEBPA* and *CEBPD* was not apparently regulated by osthole (Figure 3C). However, *CEBPB* expression increased significantly with 50 μM osthole compared to the control group (Figure 3C). Moreover, osthole increased *CEBPG* expression in a dose-dependent manner (Figure 3C). Treatment with 50 μM osthole significantly upregulated *TFE3* expression compared with the control group, whereas *NRF2* and *TFEB* expression remained unchanged (Figure 3C). Taken together, these results indicate that TFE3 may function as a potential mediator of ATF4-FGF21 signaling. Nevertheless, the induction of ATF4 by osthole cannot be accounted for by any of the currently known ATF4-regulating transcription factors.

### 2.4. ATF4 Knockdown Suppresses Osthole-Induced Fgf21 Expression

**Figure 4:**
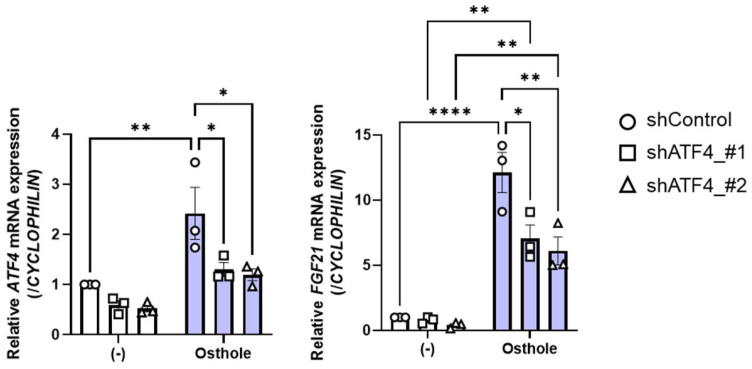
Knockdown of ATF4 suppresses osthole-induced FGF21 expression. Expression levels of ATF4 and FGF21 in HepG2 cells transfected with shATF4 plasmids and treated with 50 µM osthole or DMSO, which is indicated as (−), for 24 h. *n* = 3 per group. Data are presented as the mean ± SE. * *p* < 0.05; ** *p* < 0.01; **** *p* < 0.0001. Statistical comparisons were performed using the one-way ANOVA, followed by Tukey’s post hoc test.

To confirm the necessity of ATF4 for osthole-induced *FGF21* expression, we performed a loss-of-function analysis in HepG2 cells by knocking down ATF4 using shRNA (shATF4). The cells were transfected with two types of shRNA vector against ATF4 and then incubated for 24 h before being treated with 50 μM osthole for a further 24 h. We confirmed that both types of shATF4 significantly suppressed *ATF4* expression with and without osthole treatment (Figure 4). ATF4 knockdown efficiently suppressed osthole-induced *FGF21* expression (Figure 4), demonstrating that ATF4 plays a critical role in osthole’s effects on FGF21 expression.

## 3. Discussion

FGF21 has been a major focus in metabolic research due to its potential therapeutic applications. Scientists are actively investigating compounds that can enhance FGF21 expression to improve metabolic health, particularly in conditions like obesity, diabetes, and muscle metabolism. Our study revealed that osthole, which is included in *Cnidium monnieri*, would be a novel compound to increase *FGF21* expression in HepG2 cells, which belong to a human hepatoma cell line, by activating ATF4.

In this study, we isolated osthole from the WAKANYAKU library as a candidate to increase *FGF21* expression. Osthole reduces triglyceride (TG) and free fatty acid levels in serum and hepatic tissues in high-fat-induced fatty liver rat and quail models or alcoholic-induced fatty liver rat and mouse models [27,28,29]. These effects are due to increments in the levels of mRNA and protein of PPARα and PPARγ in the liver, which are caused by osthole [30,31]. In cultured hepatocytes, osthole suppresses oleic acid/lipopolysaccharide-induced lipid accumulation and the inflammatory response by activating PPARα [32]. Taken together, osthole is thought to be a dual PPARα/γ agonist. However, in our study, osthole did not increase PPARα expression; therefore, we could not confirm that osthole activates PPARα.

PPARα [12], along with CREBH [13], is an important regulator of *FGF21* mRNA, which is elevated in the liver during fasting. PPARα was not upregulated by osthole whereas CREBH was upregulated. ChREBP, which is also regulated during feeding [14], was unchanged. *FGF21* expression is induced by ATF4 under conditions of protein restriction and amino acid deficiency. Although ATF4 belongs to this same transcription factor family, other ER stress–responsive transcription factors, including CREBH [33], ATF6 and XBP1s [18], can also induce *FGF21* expression. In this study, osthole was found to upregulate the expression of *CREBH*, *ATF4*, and *XBP1s*, suggesting that osthole may activate ER stress signaling, thereby contributing to increased *FGF21* expression. Osthole was found to increase the gene expression of *ATF3* and *ASNS*, which are target genes of ATF4. Therefore, it is important to note that osthole activates ATF4 signaling. Osthole-induced *FGF21* expression was suppressed by *ATF4* knockdown, confirming ATF4’s essential role in osthole’s mechanism of action. Treatment with 50 μM osthole increased the expression of *ATF4*, *CREBH*, and *XBP1s*. These results imply that osthole-induced *FGF21* expression requires the activation of several transcription factors. However, ATF4 knockdown experiments revealed that the reduction in ATF4 expression was comparable to the decrease in osthole-induced *FGF21* expression. If so, a complete knockdown of ATF4 would be expected to almost entirely abolish osthole-induced *FGF21* expression. This outcome would suggest that ATF4 is essential for this process, while CREBH and XBP1s are dispensable, which is contradictory to their observed induction. Therefore, we propose that the osthole–FGF21 signaling pathway is initiated by ATF4 and subsequently amplified through a positive feedback loop involving the activation of CREBH and XBP1s.

ATF4 translation is known to be activated by PERK–eIF2α signaling [15]. However, osthole did not alter the levels of phosphorylated eIF2α, indicating that osthole-induced ATF4 activation is not mediated through this pathway. At the transcriptional level, TFE3, TFEB, and NRF2 are reported activators of *ATF4* expression [21,22], whereas the C/EBP family functions as repressors [23,24]. The observed changes in the expression of these genes alone cannot fully explain the mechanism. Although osthole increased *TFE3* expression, no studies have reported that TFE3 directly regulates *FGF21* expression. Thus, the precise mechanism by which osthole upregulates *ATF4* remains unclear. TFE3 is a master regulator of autophagy [34], and given that ATF4 controls amino acid metabolism, we speculate that there may be a functional relationship between them. Further studies will be necessary to clarify whether TFE3 serves as a key mediator of ATF4 upregulation. Importantly, TFE3-mediated activation of autophagy contributes to the improvement of lifestyle-related diseases. In addition, TFE3 enhances insulin signaling and suppresses lipid synthesis [35], thereby ameliorating lifestyle-related diseases. These effects of TFE3 may underlie, at least in part, the therapeutic potential of osthole in lifestyle-related diseases.

Previous studies have reported that several herbal-derived compounds can induce *FGF21* expression through distinct signaling pathways. For instance, berberine, a bioactive constituent of *Coptis chinensis* and *Berberis* spp. [36], as well as *Kursi Wufarikun Ziyabit*, a traditional herbal formulation containing *Geranium collinum* and *Hypericum scabrum* [37], enhance *FGF21* expression via activation of AMP-activated protein kinase. In addition, wogonin has been shown to induce *FGF21* through ATF4-dependent signaling [38]. In this context, our study identifies osthole, a bioactive component of herbal medicines, as a novel activator of FGF21, thereby expanding the repertoire of phytochemicals capable of modulating FGF21 and providing new insight into the diversity of upstream regulatory mechanisms.

In conclusion, although osthole has been reported to activate PPARα in the liver of mice and in rat hepatocytes [31,32], its role in regulating *FGF21* expression had remained unclear. Our data demonstrates that osthole does not induce *PPARA* expression in HepG2 cells but instead stimulates *FGF21* expression through an ATF4-dependent pathway. Using HepG2 cells as a model of hepatic FGF21 regulation, we identify a previously unrecognized mechanism by which osthole activates FGF21. Given the established metabolic benefits of FGF21, these findings highlight osthole as a promising candidate for the prevention and treatment of lifestyle-related metabolic diseases.

## 4. Materials and Methods

### 4.1. Cell Culture

HepG2 cells (RGB1648) were provided by RIKEN BioResource Research Center, Ibaraki, Japan. HepG2 cells were cultured at 37 °C in a 5% CO_2_ environment in DMEM medium (Nacalai Tesque, 08458-45, Kyoto, Japan) supplemented with 10% fetal bovine serum (CORNING, 35-079-CV, Corning, NY, USA), 100 U/mL penicillin, and 100 μg/mL streptomycin (Nacalai Tesque, 09367-34, Kyoto, Japan). Cells were treated with 10 µM or 50 µM osthole (Tokyo Chemical Industry Co., Ltd., O0426, Tokyo, Japan).

### 4.2. Plasmids

pGL3-FGF21 contained −2 kbp to −40 bp of the mouse *Fgf21* promoter [13]. pGL3-ATF4 contained −0.5 kbp to −100 bp of the mouse *Atf4* promoter [38]. Control shRNA: shControl (pDNA(VB010000-0023jze): 5′-CCTAAGGTTAAGTCGCCCTCG-3′) and human ATF4 shRNA vectors: shATF4 1 (pDNA(VB900147-1829hks) 5′-CATGATCCCTCAGTGCATAAA-3′ and shATF4 2 pDNA(VB900147-1830wwp) 5′-CCTAGGTCTCTTAGATGATTA-3′) were purchased from Vector Builder (Chicago, IL, USA). These plasmids were transfected into HepG2 cells with FuGENE6 (Promega, E2691, Madison, WI, USA).

### 4.3. Luciferase Analysis

HepG2 cells were transfected with the indicated luciferase vector and pRL-CMV (Promega, E2261, Madison, WI, USA), as a reference, using FuGENE6 (Promega, E2691, Madison, WI, USA). After 24 h of incubation, cells were treated with 10 µM or 50 µM osthole for 24 h. Firefly and Renilla luciferase activity was measured using the Dual-Luciferase^®^ Reporter Assay System (Promega, E1910, Madison, WI, USA). Firefly luciferase activities were normalized to Renilla luciferase activities.

### 4.4. Quantitative Polymerase Chain Reaction (qPCR)

Total RNA was isolated from collected cells using Sepasol^®^-RNA I Super G (Nacalai Tesque, 09379-55, Kyoto, Japan) as per the manufacturer’s protocol. All samples passed the RNA quality control as assessed on NanoDrop Lite Spectrophotometer (Thermo Fisher Scientific, Waltham, MA, USA). Total RNA (1 µg) was reverse-transcribed using ReverTra Ace^®^ qPCR RT Master Mix (Toyobo, FSQ-201, Osaka, Japan). qPCR was performed on a CFX Connect Real-Time PCR Detection System (Bio-Rad, Hercules, CA, USA) using THUNDERBIRD^®^ Next SYBR^®^ qPCR Mix (Toyobo, QPX-201, Osaka, Japan). Samples were quantified via the 2^−ΔΔCt^ method and normalized to Cyclophilin levels to quantify the relative mRNA expression. The qPCR primer sequences are outlined in Table 1.

### 4.5. Western Blotting

Cells were lysed in the lysis buffer containing 10 mM Tris-HCl, 100 mM NaCl, 1 mM EDTA, 1 mM EGTA, 1 mM NaF, 20 mM Sodium pyrophosphate, 2 mM Sodium orthovanadate, 0.1% SDS, 0.5% Sodium deoxycholate, 1% Triton X-100, 10% Glycerol, and Complete Protease Inhibitor (Roche, 4693116001, Basel, Switzerland). The total cell lysates were subjected to 10% sodium dodecyl sulfate-polyacrylamide gel electrophoresis (SDS-PAGE) and transferred to Immobilon-P PVDF membranes (Millipore, IPVH00010, Burlington, MA, USA). The membranes were blocked with 5% skim milk in TBS-T. The membranes were incubated with anti-ATF4 (Santa Cruz, sc-390063, 1:000, Dallas, TX, USA), anti-phosho-eIF2α (Cell Signaling Technologies, 3298, 1:1000, Danvers, MA, USA), anti-eIF2α (Cell Signaling Technologies, 5324, 1:1000, Danvers, MA, USA), and anti-α-tubulin (Santa Cruz Biotechnology, sc-32293, 1:1000, Dallas, TX, USA) antibodies. Alpha-tubulin was used as an internal control. After washing, the membranes were incubated with horseradish peroxidase-conjugated mouse IgG (Cell Signaling Technologies, 7076, 1:5000, Danvers, MA, USA) and rabbit IgG (Cell Signaling Technologies, 7074, 1:5000, Danvers, MA, USA). The immunoreactive bands were detected by ChemiDoc XRS^+^ (BioRad, Hercules, CA, USA) using ImmunoStar LD (WAKO, 290-69904, Osaka, Japan). The immunoreactive band intensity was quantified using Image Lab software 6.1 (BioRad, Hercules, CA, USA).

### 4.6. Statistical Analysis

Continuous data were expressed as the mean ± standard error (SE), while categorical data were expressed as frequencies with percentages. Comparisons of continuous data between two groups were performed using the unpaired Student’s *t*-test. Comparisons of continuous data among multiple groups were performed using the one-way ANOVA, followed by Tukey’s post hoc test. All statistical analyses were performed using GraphPad Prism 10 (GraphPad Prism Software, San Diego, CA, USA). *p*-values < 0.05 were considered statistically significant.

## 5. Conclusions

In this study, we identified osthole as a phytochemical that induces *FGF21* expression, providing a basis for exploring its potential therapeutic relevance to lifestyle-related metabolic diseases. Although our results demonstrate that osthole primarily activates *FGF21* expression through an ATF4-dependent pathway, potential interactions with other transcription factors, including CREBH and XBP1s, were suggested but not mechanistically resolved. In particular, the molecular basis by which osthole, together with ATF4, activates CREBH and XBP1s remains unclear. Elucidating the cooperative or hierarchical regulatory mechanisms among these transcription factors will be an important focus of future studies. In addition, the ATF4–FGF21 axis was mainly examined using HepG2 cells, and it remains to be determined whether a similar regulatory mechanism operates in other hepatic cell models or in extrahepatic tissues. Finally, this study was largely based on in vitro analyses. Further investigation is required to determine whether osthole induces hepatic *FGF21* expression and improves metabolic phenotypes in lifestyle disease models in vivo, as well as its effects on other tissues and potential adverse effects.

## Figures and Tables

**Table 1:** Primers used for real-time PCR analysis.

Gene Name	Fwd	Rv
*ASNS*	CTGTGAAGAACAACCTCAGGATC	AACAGAGTGGCAGCAACCAAGC
*ATF3*	CGCTGGAATCAGTCACTGTCAG	CTTGTTTCGGCACTTTGCAGCTG
*ATF4*	CCAACAACAGCAAGGAGGAT	AGGTTCATCTGGCATGGTTTC
*ATF6*	CAGGCAGTACCAACGCTTATGCC	GCAGAACTCCAGGTGCTTGAAG
*CEBPA*	AGGAGGATGAAGCCAAGCAGCT	AGTGCGCGATCTGGAACTGCAG
*CEBPB*	AGAAGACCGTGGACAAGCACAG	CTCCAGGACCTTGTGCTGCGT
*CEBPD*	TCCGGCAGTTCTTCAAGCAGCT	GAGGTATGGGTCGTTGCTGAGT
*CEBPG*	GCTTACAGCAGGTTCCTCAGCT	CGTTGCCGATACTCGTCACTGT
*CHOP*	GGTATGAGGACCTGCAAGAGGT	CTTGTGACCTCTGCTGGTTCTG
*ChREBP*	GCGTTTTGACCAGATGCGAGAC	CGTTGAAGGACTCAAACAGAGGC
*CREBH*	CCTCTGTGACCATAGA	ACGGTGAGATTGCATC
*FGF21*	ACTCCAGTCCTCTCCT	AGTGGAGCGATCCATA
*NRF2*	CACATCCAGTCAGAAACCAGTGG	GGAATGTCTGCGCCAAAAGCTG
*SLC7A11*	TCCTGCTTTGGCTCCATGAACG	AGAGGAGTGTGCTTGCGGACAT
*TFE3*	GATCATCAGCCTGGAGTCCAGT	AGCAGATTCCCTGACACAGGCA
*TFEB*	CCTGGAGATGACCAACAAGCAG	TAGGCAGCTCCTGCTTCACCAC
*XBP1s*	AGCTTTTACGAGAGAAAACTCA	GCCTGCACCTGCTGCG
*CYCLOPHILIN*	GGCAAATGCTGGACCCAACACA	TGCTGGTCTTGCCATTCCTGGA

## Data Availability

The original contributions presented in this study are included in the article and Supplementary Materials. Further inquiries can be directed to the corresponding author.

## Supplementary Materials

The following supporting information can be downloaded at https://www.mdpi.com/article/10.3390/ijms27021003/s1.

## Abbreviations

The following abbreviations are used in this manuscript:

FGF21	Fibroblast growth factor 21
ATF4	activating transcription factor 4
MAFLD	metabolic dysfunction-associated fatty liver disease
MASH	metabolic dysfunction-associated steatohepatitis
PPAR	peroxisome proliferator-activated receptor
PGC-1α	peroxisome proliferator-activated receptor gamma coactivator 1-alpha
CREBH	cyclic adenosine monophosphate-responsive element-binding protein H
ChREBP	carbohydrate-responsive element-binding protein
NRF2	nuclear factor erythroid 2-related factor 2
ER	endoplasmic reticulum
XBP1s	X-box binding protein 1s
ISR	Integrated Stress Response
PERK	PKR-like ER kinase
TFE3	transcription factor E3
CEBPB	CCAAT/enhancer-binding protein β
ASNS	asparagine synthetase
CHOP	CCAAT/enhancer-binding protein homologous protein
SLC7A11	solute carrier family 7 member 11
