# Supplementary material for: Osthole Activates FGF21 Expression by Mediating Activation of ATF4 in Human Hepatocyte HepG2 Cells
Source: Int J Mol Sci. 2026 Jan 19;27(2):1003. doi: 10.3390/ijms27021003 (PMC12842164; doi:10.3390/ijms27021003)
Supplement: Supplementary file 1 [file ijms-27-01003-s001.zip › ijms-4073883-supplementary.pdf]

Supplementary material

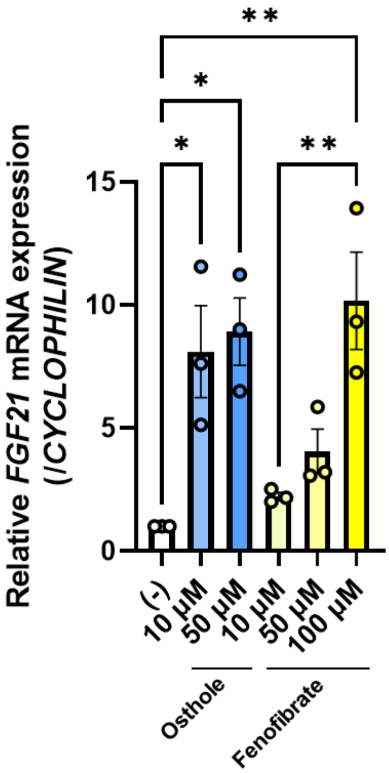

Figure S1. FGF21 gene expression levels in HepG2 cells treated with 10 μM or 50 μM osthole or 10 μM, 50 μM or 100 μM fenofibrate (SIGMA, F6020) for 24 hours. n = 3 per group. Data are presented as the mean ± SE. *P < 0.05; **P < 0.01. Statistical comparisons were performed using the one-way ANOVA, followed by Tukey's post hoc test.
